# Three-dimensional analysis of posed smile in adults: A scoping review

**DOI:** 10.1016/j.jds.2023.10.021

**Published:** 2023-11-30

**Authors:** Patcharamas Banditsaowapak, Johnson Hsin-Chung Cheng, Daniel De-Shing Chen, Michelle Yuching Chou

**Affiliations:** a School of Dentistry, College of Oral Medicine, Taipei Medical University, Taipei, Taiwan; b Orthodontic Division, Department of Dentistry, Taipei Medical University Hospital, Taipei, Taiwan; c Department of Developmental Biology, Harvard School of Dental Medicine, Boston, MA, USA

**Keywords:** Smile, Three dimensional, Stereophotogrammetry, Facial scanner, Structured light

## Abstract

This scoping review investigated the evidence on the three-dimensional analysis of a posed smile in adults to discover any research gaps in this research area. Electronic searches of articles written in English were performed using the four databases of Embase, PubMed, Springer, and Web of Science with publications from 2010 to 2023. Reference lists were also manually searched to identify additional studies. The results showed that 13 cross-sectional descriptive studies from Asia, Europe, North and South America met our inclusion criteria. Studies mainly focused on linear and angle measurement for resting and smiling faces and landmark movement from resting to smiling. Most studies conducted analysis of smiles stratified by sex, ethnicity, smile type, dental occlusion, skeletal pattern, and age. Two studies compared smiling with the resting position and one study compared the attractive smiling group with the ordinary group. Our scoping review revealed the insufficiency of some measurement methods, such as those employing area, volume, and soft tissue thickness. Furthermore, few studies were conducted in Asian populations, and comparisons of various smile types, overjet types, horizontal skeletal patterns, and comparisons of smiles between people with untreated normal occlusion and those who had been orthodontically treated were lacking.

## Introduction

Facial attractiveness in modern society assists in enhancing self-esteem and promoting relationships, employment opportunities, and business success.^1^ The mouth area, particularly smile characteristics, has been analyzed in terms of facial attractiveness.^2^, ^3^, ^4^ At present, smile esthetics tend to be people’s principal concern when they pursue dental treatment.^5^

Rubin was the first to examine the anatomical analysis of normal smile, and he divided it into three types: the Mona Lisa, the canine, and the full denture smile. He pointed out that changes in the anatomy of the muscle, soft tissue, and bones can affect the smile.^6^ Smile can also broadly be divided based on neurological control into “unposed” smile, which is involuntary related to emotion induced by enjoyment, and “posed” smile, which is voluntary and not related to emotion. It might be a formal welcome, a way to placate someone, or an effort to project confidence.^7^^,^^8^ Because posed smile is repeatable over time, it has been mainly studied by orthodontists.^4^^,^^9^^,^^10^ For the treatment of patients with facial paralysis as well as for the diagnosis and evaluation of orthodontic treatment, a precise comprehension of the normal posed smile and precise quantitative measurements are required.^8^^,^^9^

Quantitative smile analysis has been conducted. Hulsey^10^ quantified the relationship of lips and teeth displayed when smiling and compared the smiles of people with untreated normal occlusion and those who had been orthodontically treated. He calculated the smile line ratio, smile symmetry ratio, and buccal corridor ratios and measured upper lip height curvature. Other smile measurements have been evaluated in numerous studies, including the interlabial gap, intercommissural width, smile index, incisal exposure, gingival display, lower lip to incisor, and upper and lower vertical lip thickness.^4^^,^^7^^,^^11^, ^12^, ^13^, ^14^, ^15^

Many methods have been used to evaluate smile esthetics, such as photographs, radiographs, model scanning, three-dimensional (3D) surface imaging, video, and clinical assessment.^16^ Two-dimensional (2D) approaches cannot precisely evaluate the complicated 3D soft tissue of the face.^17^ Studies reported that 3D orofacial imaging techniques can be used in daily dental practice, with digital animated models providing possibilities for quantitative diagnosis in three dimensions and the evaluation of treatment outcomes.^18^

Most smile analysis research, including the aforementioned studies, has employed 2D techniques. Few studies applied 3D techniques, and this scoping review was thus conducted to investigate the 3D of a posed smile in adults as well as to discover any research gaps to benefit people interested in researching smiles using 3D techniques that are increasing.

## Materials and methods

### Search strategy

A literature review was performed following the checklist and definitions of the PRISMA extension for scoping review.^19^ Electronic searches of articles written in English were performed using the four databases of Embase, PubMed, Springer, and Web of Science with publications from 2010 to 2023. Reference lists were also manually searched to identify additional studies. The keywords used for the search were as follows: ((smile[MeSH Terms]) AND ((3D) OR (three dimensional) OR (Three dimensions) OR (3 dimensions) OR (stereophotogrammetry) OR (photogrammetry) OR (facial scanner) OR (facial scan) OR (structured light)).

### Study selection as well as inclusion and exclusion criteria

The inclusion criteria were as follows: studies must have (1) reported measurements and conducted analyses of posed normal smile; (2) involved a group of adult participants with no obvious skeletal discrepancy, congenital defect, maxillofacial trauma, facial paralysis or history of orthodontic treatment; and (3) used a 3D camera. The exclusion criteria were as follows: studies that were (1) literature reviews; (2) scoping reviews; (3) systematic reviews and meta-analyses; or (4) case reports.

Two impartial reviewers independently extracted the data, and any discrepancies were settled with the help of a third reviewer. The information that was taken from the study’s data included the study’s participants, measurements, comparisons, results, and conclusions.

## Results

### Selection of sources of evidence

**Fig. 1:**
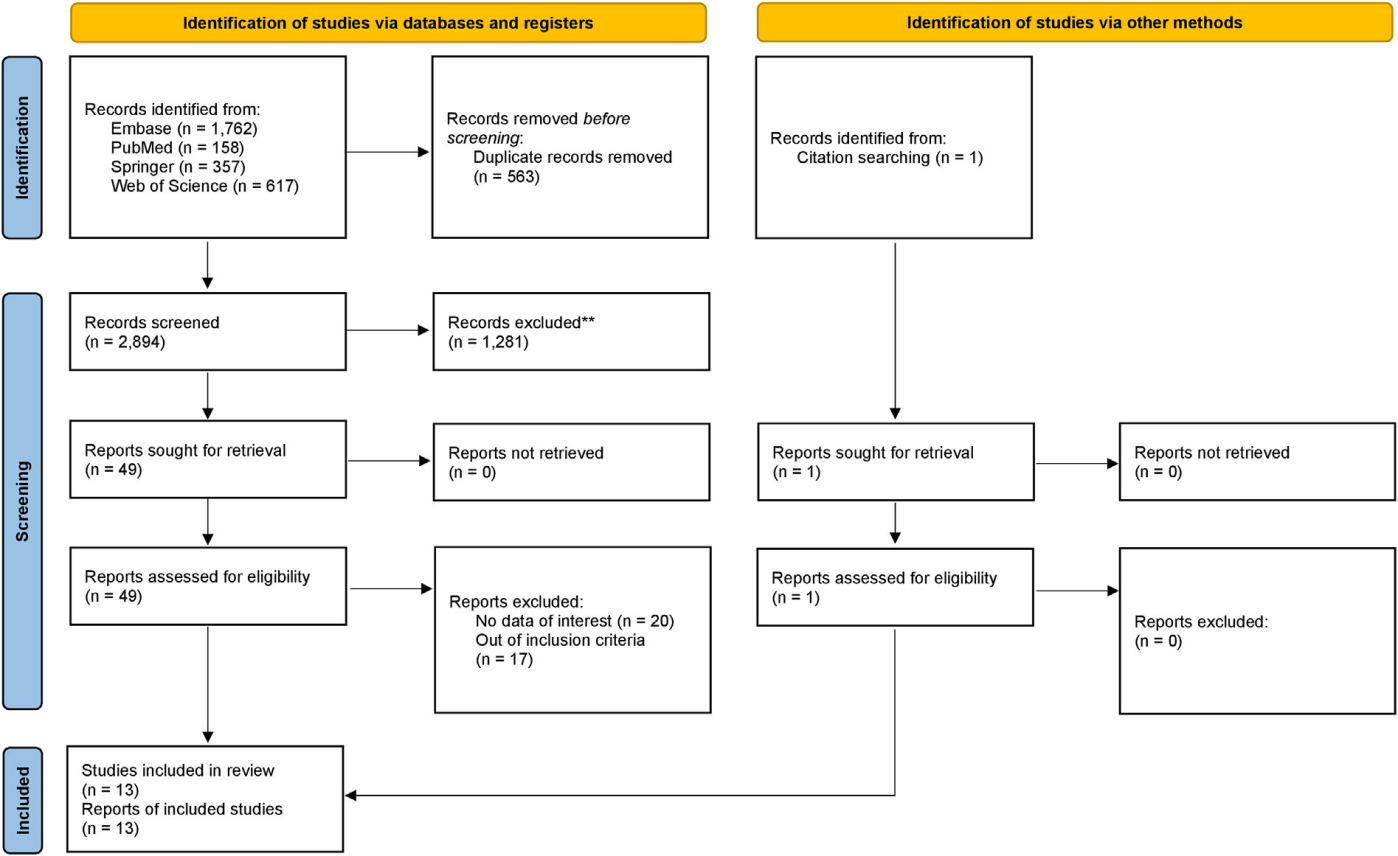
PRISMA 2020 flowchart for systematic reviews.

A total of 2894 records were collected through the electronic search. After duplicates were removed, 2331 records were identified. According to the PRISMA statement, we screened the abstracts and assessed the available full-text articles based on the eligibility criteria. Including one record retrieved in the manual search, 13 articles were examined in this scoping review. The process of the selection of sources of evidence is detailed in the PRISMA flowchart (Fig. 1).

### Characteristics of sources of evidence

The characteristics of the included studies are described in Table 1. All studies had a cross-sectional descriptive design. Four studies were conducted in Europe, three in North America (the United States), three in Asia, and one each in South America and Turkey. The participants have an average age ranging from 18.62 to 34 years or between 15 and 60 years, except for participants age over 60 years in two studies.^20^^,^^21^

**Table 1:** Summary of descriptive characteristics of included studies.

Author(s)/year	Study design (Country)	Participants			Measurements		Comparisons (Independent variables)	Results	Conclusion
		M	F	Total	Types	Details			
Campbell et al., 2012	Cross-sectional (Ireland)	–	40	40	Distance of landmark displacement from resting to smiling	The root mean square (RMS) difference of 26 facial landmark points across three planes between the resting and smiling positions (natural smile and maximal smile) were calculated	Dental occlusion and skeletal pattern (overjet: 2–4 mm vs. 6–10 mm)	For both natural and maximal smile, the average movement of the landmarks was greater in the 2–4-mm overjet group than in the 6–10-mm overjet group	A large overjet could noticeably influence the range of smile movement
Ceinos et al., 2017	Cross-sectional (France)	28	22	50	Linear ratio during smiling	- Facialproportions (Gla-SN/SN-Me) -IE-Me/SN-IE (incisal edge of the maxillary central incisor–lower border of the chin/subnasale–incisal edge of the maxillary central incisor) -EnCr-EnCl/ExCr-EnCr (right endocanthion–left endocanthion/right exocanthion–right endocanthion) -IE-DCr/DCr-LCr (incisal edge of the maxillary central incisor–distal edge of the right canine/distal edge of the right canine–right commissure) -CLr-CLl/DCr-DCl (right commissure–left commissure/distal edge of the right canine–distal edge of the left canine)	Participant vs. golden ratio (1.618)	-IE-Me/SN-IE was 1.693 ± 0.190, which was different from the golden ratio -EnCr-EnCl/ExCr-EnCr was 1.582 ± 0.173 -IE-DCr/DCr-LCr was 1.670 ± 0.355 -CLr-CLl/DCr-DCl was 1.602 ± 0.136	Only the vertical ratio (IE-Me/SN-IE) differed from the golden ratio, whereas the horizontal ratios were similar to the golden ratio
Demir and Baysal, 2020	Cross-sectional (Turkey)	60	60	120	-Linear and angle measurement in the resting and smiling positions -Linear ratio during smiling -Landmark displacement across three planes from resting to smiling	Linear measurements: -Nasal arch length -Nasal projection -Nasal width -Upper lip length -Upper lip vermillion length -Lower lip vermillion length -Interlabial gap -Philtrum width -Intercomissural width Linear measurements during smiling only: -Maxillary incisor and gingival display -Maxillary intercanine width -Buccal corridors Angle measurements: -Upper lip angle -Lower lip angle -Nasal protrusion angle -Nasal aspect Ratios during smiling only: -Smile index -Buccal corridor ratio	-Dental occlusion and skeletal pattern (normal vs. increased vertical facial height) -Sex (male vs. female)	When smiling, the vertical group had shorter upper lips, higher upper lip elevation, and less horizontal commissure movement than the normal group. The interlabial gap was larger in both the resting and smiling positions. The smile index, nasolabial fold movement, and lip angles exhibited significant differences between the two groups. In terms of sex, mouth width during smiling was larger for women than for men in the vertical group, and nasolabial fold landmark movement was greater in women than in males in the normal group	Differences were observed between two skeletal patterns during smiling. Taking these variations into account allows for more accurate diagnosis and treatment planning
Jandová and Urbanová, 2018	Cross-sectional (Czech Republic)	10	10	20	Surface comparison method based on aligned 3D meshes and the closest point-to-point distances from resting to smiling	Calculations: -RMS −75th percentile	Sex (male vs. female)	The difference was not significant between men and women in wild smile position	Men exhibited more facial movements than women. However, the difference was not significant in a wild smiling position
Li et al., 2019	Cross-sectional (China)		50	50	-Linear measurement in the resting and smiling positions -Linear ratio during smiling -Landmark displacement across three planes from resting to smiling	Linear measurements: -Interlabial gap -Intercommissural width Ratio during smiling only: -Smile index Landmark displacement: -Commissure or cheilion (right and left) -Lip superior -Lip inferior	Dental occlusion and skeletal pattern (normal vs. increased vertical facial height vs. horizontal facial height)	Smile indices were ordered from high to low as follows: horizontal > average > vertical. The interlabial gap exhibited significant differences among the three groups. The upper lip landmarks of the vertical group became more displaced along the z-axis than those of the other groups. No difference was observed in lower lip landmarks in any direction. The variations of the lip landmarks were ranked from high to low as follows: horizontal > average > vertical	The characteristics of a smile vary depending on skeletal patterns. Upper lip displacement was impacted but not lower lip displacement by the increased vertical skeletal pattern
Li et al., 2023	Cross-sectional (China)	–	60	60	Landmark position	The anteroposterior position of maxillary incisor landmark (mFA) relative to the following vertical line: -The vertical line passed through soft tissue subnasale (SSn) -The vertical line passed through glabella -The vertical line passed through the midpoint between the most rounded contour of the forehead and glabella (FFA) -The modified of FFA vertical line in case of the forehead inclination is more than 7° (GALL)	Attractive score (attractive smiling female vs. ordinary female)	For 90° profile, there were significant differences between study group and control group in the distances of mFA-SSn (−6.31 ± 0.96 vs. −2.96 ± 1.94 mm), mFA-Glabella (−4.97 ± 1.54 vs. −1.25 ± 2.64 mm), mFA-FFA (−1.35 ± 2.10 vs. 1.74 ± 2.89 mm), and mFA-GALL (−4.48 ± 1.87 vs. −0.70 ± 2.65 mm). For 45° profile, there were significant differences between study group and control group in the distances of mFA-SSn (−4.46 ± 0.68 vs. −2.07 ± 1.35 mm), and mFA-Glabella (−4.46 ± 0.68 vs. −2.07 ± 1.35 mm)	The mFA-Glabella and mFA-SSn distances had smaller standard deviations than the other measurements for 90° profile and those distances in study group had smaller standard deviations than in control group for 45° profile. As a result, they concluded that the mFA-SSn and mFA-Glabella distances are esthetically important factors in female profiles.
Masoud et al., 2017	Cross-sectional (United States)	25	24	49	-Landmark position -Linear measurement projected across three planes in the resting and smiling positions	Landmark position: right and left alar curvature, subnasale, subspinale, labrale superius, labrale inferius, sublabiale, and pogonion Linear measurements: -Maxillary right and left incisal display during smiling -Maxillary right and left gingival display during smiling -Smile width -Upper lip length	Sex (male vs. female)	Ratios and angular measurements exhibited no significant difference between sexes except in relation to the occlusal plane, which was higher in women than in men. Men had more proclined upper incisors (20° vs. 16°) and more retroclined lower incisors (27° vs. 31°) than women. Men exhibited greater smile width (72.11 vs. 61.89 mm) than women	Men and women had different characteristics. Clinicians can use these characteristics to distinguish dentofacial norms for orthodontic diagnosis
Parra et al., 2019	Cross-sectional (Chile)	10	10	20	Linear and angle measurement in the resting and smiling positions	Linear measurements: -Length of the alar base Angle measurements: -Nasolabial angle	Resting vs. smiling	The average length of the alar base was 34.3 ± 2.6 mm during resting, and 39.1 ± 2.9 mm during smiling. The average nasolabial angle was 104.6° ± 9.6° during resting and 105.4° ± 14.3° during smiling. The distance between the alar base and nasolabial angle increased by an average of 4.83 mm and 0.8° consecutively from resting to smiling	The nasolabial angle was unchanged, whereas the length of the alar base changed significantly during smiling
Pucciarelli et al., 2018	Cross-sectional (Italy)	10	10	20	Surface comparison method based on aligned 3D meshes and the closest point-to-point distances from resting to smiling	Calculations: -RMS -Labial surface areas in the resting and smiling positions -Percentage modification for each smile type	-Smile types (Mona Lisa smile vs. canine smile vs. full-denture smile) -Sex (male vs. female)	RMS values gradually enlarged from the Mona Lisa to full-denture smile. The differences were significant for facial and labial models among the three types of smiles. Sex had no significant effect on RMS value. Labial surface areas and percentage modification exhibited significant differences in relation to sex and smile type	RMS values varied greatly depending on the type of smile. In terms of sex and smile type, Differentiations in the labial surface areas and percentage modification were statistically significant.
Sawyer et al., 2010	Cross-sectional (United Kingdom)	38	33	71	-Distance (vector) of landmark displacement across three planes from resting to smiling -Distance and angle of landmark displacement across the 2D and 3D planes from resting to smiling	Landmarks: -Cheilion (right and left) -Labiale superius -Upper mid-lateral lip (right and left) -Labiale inferius -Lower mid-lateral lip (right and left) -Nasolabial fold (right and left)	- sex (male vs. female)	The average distances and angles of landmark movement when smiling in the 3D plane were as follows: Cheilion: 16.6 mm at 31°; labiale superius: 8.2°; upper mid-lateral lip: 10.5°; labiale inferius: 5.3°; lower mid-lateral lip: 7.8°; and nasolabial fold: 12.6°. Although men exhibited greater movement of landmarks than women, no significant difference was observed	A quantitative approach was proposed for measuring smiles to evaluate the result of different surgical operations. No significant difference in landmark movement in terms of sex was observed
Souccar et al., 2019	Cross-sectional (United States)	205	189	394	Linear measurement in the resting and smiling positions	Resting: -Upper lip length -Width of mouth -Lower lip length Smiling: -Upper lip length -Width of mouth -Lower lip length -Gingival display -MI crown length	-Sex (men vs. women) -Ethnicity (African American vs. Caucasian) -Age (20–30, 30–40, 40–50, 50–60, and older than 60 years)	All dimensions were larger in men than in women. Except for the length of the upper lip in men, all measures were greater in African Americans than in Caucasians. Gingival and maxillary incisor display during smiling decrease as age increases	Sex, ethnicity, and age have an effect on smile dimensions
Tanikawa et al., 2019	Cross-sectional (Japan)	–	130	130	-Surface comparison method based on aligned 3D meshes and the closest point-to-point distances -Curving lines -Inter-landmark distances projected across three planes -Linear ratios	-vectors connecting the older group’s average mesh points to the younger group’s -5categories of curving lines: inter-landmark contours, sagittal sections, axial sections, facial outlines, and supraorbital ridge outlines -Inter-landmark distances: nasal bridge length, maxilla height, lower face height, upper lip vermillion height, lower lip vermillion height, mandible height -Ratios: total midface-lower face height, midface-lower face height index, lower face-face height, mandible-upper face height, mandible-lower face height, chin-lower face height, facial index, upper face index, face height-mandible width index, mandibular index, mouth-face index	-Resting vs. smiling -Age (18–32 vs. 55–65 years)	-Surface comparison analysis: The mouth corners of the older group at rest and smile were narrower along the x-axis. The older group had lower-positioned cheeks and chin vertical positions during rest and smile, according to the y-axis. When the older group was at repose, their mouth corners and lower lips were situated in more inferior positions, and when they smiled, their upper lips were situated in more inferior positions. Along the z-axis, when an older group was at rest and smiling, the nasal bridge and infraorbital areas were more protuberant along the z-axis. At rest, the older group had more retruded mouth corners. -Inter-landmarks distances and ratios at rest in the order group: sagging skin in the cheeks of the zygomatic region and facial shape, protrusion of the nasal wing, wider lower face, mouth placed lower on the face, and flabby cheeks at the level of the lips and chin. When smiling, the order group features the following: a lower-positioned mouth corner, a convex subnasal profile, sagging skin at the mandibular angle, and a larger mouth protrusion. -Landmark displacement analysis: In the older group, 66 variables shown significant shifts from resting to smiling. Significant changes in the younger group were seen in 144 variables. -Discriminant analysis for resting vs. smiling: From rest to smile, the younger group had a large retrusive movement of the lip commissure and upper lip, a decrease in the labio-mental fold, a retrusive movement of the lower lip, a decrease in protrusion at the level of the lower lip and at the chin, and an increase in facial width. In contrast, the older group had a significant decrease in lower lip height but increase in cheek protrusion, and retrusive movement of nasal ala. Significant moderate correlations and weak correlations were discovered between vertical cephalometric skeletal measurements and soft tissue smiling characteristics in the study. Along the y- and z-axis, significant multiple regression models were obtained for the lower lip, intercommissural width and smile index.	There were substantial age-related 3D face alterations in the creation of facial expressions, and the transition from resting to smiling produced less soft tissue movement in the older group than in the younger group. Facial morphology differed significantly between younger and older respondents in both facial expressions, with the older group’s morphology during smiling being more difficult to distinguish from the morphology at rest.
Toth et al., 2016	Cross-sectional (United States)	–	110	110	-Linear measurement in the smiling positions -Linear ratio during smiling -Landmark displacement across three planes from resting to smiling	Linear measurement in the smiling positions: -Interlabial gap -ntercommissural width Linear ratio during smiling: Smile index Landmarks: -Lip superior -Lip inferior -Commissure or cheilion (right and left) -Crista philtre (right and left)	Dental occlusion and skeletal pattern (vertical cephalometric skeletal measurements: SN-GoGn, anterior facial height, and lower and upper facial height proportion	Significant moderate correlations and weak correlations were discovered between vertical skeletal measurements and soft tissue smiling features in the study. Along the y- and z-axis, for the lower lip, intercommissural width and smile index all had significant multiple regression models.	They concluded that as SN-GoGn and anterior face height increased the interlabial gap increased while the smile index decreased. Certain hard tissue cephalometric measurements were shown to be related to the width of the smile as well as lower lip motions.

### Synthesis of results regarding type of measurement (dependent variable)

#### Linear and angle measurement of resting and smiling faces

Five studies reported only linear measurement of resting and smiling faces,^20^, ^21^, ^22^, ^23^, ^24^ and two studies reported both linear and angle measurements.^25^^,^^26^ Maxilla height, lower face height, mandible height, nasal bridge length, width of the mouth (intercommissural width), upper lip length, upper lip vermillion length, lower lip length, and lower lip vermillion length comprise most of the linear dimensions measured at resting and smiling positions. However, the interlabial gap, gingival display, maxillary incisor display, and maxillary intercanine width, are also measured at the smiling position. The angle measurements used in these studies are the nasolabial angle, upper lip angle, and lower lip angle.

#### Curving line

Only one study by Tanikawa et al.^21^ reported the curving lines. They extracted 142 measurements to describe five categories of curving lines: inter-landmark contours, sagittal sections, axial sections, facial outlines, and supraorbital ridge outlines. Their curving lines in five categories are composed of many cross-sectional lines and angles of the 3D image surface projected across three planes.

#### Landmark distance ratio

Four studies involved proportional measurements using only smile records.^22^^,^^24^^,^^25^^,^^27^ Li et al.^22^ and Toth et al.^24^ examined the smile index (intercommissural width/interlablal gap), whereas Demir and Baysal^25^ analyzed the smile index and buccal corridor ratio. Ceinos et al.^27^ documented measurement of the following ratios: IE-Me/SN-IE (incisal edge of the maxillary central incisor–lower border of the chin/subnasale–incisal edge of the maxillary central incisor); IE-DCr/DCr-LCr (incisal edge of the maxillary central incisor–distal edge of the right canine/distal edge of the right canine–right commissure), and CLr-CLl/DCr-DCl (intercommissural width/distal edge of the right canine–distal edge of the left canine).

One study involved ratios during smiling and resting postures.^21^ Those ratios related to smiling include total midface/lower face height, midface/lower face height, lower face/face height, mandible/upper face height, mandible/lower face height, chin/lower face height, facial index, upper face index, face height/mandible width, mandibular index, and mouth/face.

#### Landmark position

There are two studies reporting the landmark position. Masoud et al.^23^ showed the positions of the left and right alar curvature, subspinale, subnasale, labrale superius, sublabiale, labrale inferius, and pogonion relative to the coronal and axial planes. The other, Li et al.,^28^ investigated the maxillary central incisor position relative to the coronal plane.

#### Landmark displacement from resting to smiling

Five articles reported the movement of oral landmarks related to the x-plane, y-plane, and z-plane from the resting position to the posed smiling position.^16^^,^^22^^,^^24^^,^^25^^,^^29^ The key landmarks used in these articles are the labiale superius, labiale inferius, and cheilion (right and left), and other landmarks including the subalare (right and left), subnasale, nasolabial fold (right and left), crista philtre (right and left), upper mid-lateral lip (right and left), and lower mid-lateral lip (right and left). Demir and Basal,^25^ Li et al.,^22^ and Toth et al.^24^ divided the average displacement of each landmark into x, y, and z vectors, whereas Campbell et al.^16^ calculated the distance movements averaged over landmarks across three planes. In addition to the two aforementioned measurements, Sawyer et al.^29^ further calculated the angle and distance of movement of landmarks across 2D and 3D planes.

#### Surface comparison method based on 3D meshes and closest point-to-point distances

Three studies used comparison methods based on 3D meshes and the closet point-to-point distances to analyze smiles.^21^^,^^30^^,^^31^ With this approach, 3D-averaged faces (surface shell meshes) of rest and smile postures are created and superimposed, and then the root mean square (RMS) differences of the point-to-point distances are obtained. In addition to the RMS, the 75th percentile is computed in the study of Jandová and Urbanová,^30^ whereas Pucciarelli et al.^31^ calculated labial surface areas and percentage modification in the resting and smiling positions. On the other hand, Tanikawa et al.^21^ created and superimposed the 3D-averaged faces of younger and older groups, then the vectors with x-, y-, and z-values from average mesh points of the younger group to those of the older group were calculated.

### Synthesis of the results of subgroup analysis (independent variable)

#### Sex

Seven articles compared smile measurements between men and women.^20^^,^^23^^,^^25^^,^^27^^,^^29^^,^^30^ Among them, four articles focusing on smile movement reported no statistically significant difference between sexes.^31^ However, the remaining two articles focusing on linear and angle measurement in the resting and smiling positions revealed that most of the measurements, including upper lip length, lower lip length, and width of mouth, are significantly larger for men than for women.^20^^,^^23^

#### Ethnicity

Only one study compared smile dimensions between two ethnicities, namely African American and Caucasian.^20^ The researchers concluded that, with the exception of the length of the male upper lip, all measures are significantly greater in African Americans than in Caucasians.

#### Smile type

One study analyzed three types of smiles, including the “Mona Lisa smile,” canine smile, and full-denture smile.^31^ RMS values, which are computed after the superimposition of all smile models in the resting position, gradually increase as smiles transition from Mona Lisa smiles to full-denture smiles. Statistically significant differences are noted for facial and labial models among the three smile types.

#### Dental occlusion and skeletal pattern

Four articles investigated dental occlusion and skeletal patterns.^16^^,^^22^^,^^24^^,^^25^ Demir and Basal,^25^ Li et al.^22^ and Toth et al.^24^ evaluated smile characteristics in terms of different vertical skeletal patterns. Demir and Basal^25^ and Li et al.^22^ reported that the interlabial gap and the movement of upper lip landmarks have a tendency to become larger in people with higher vertical skeletal patterns. The smile index exhibits the opposite results. Toth et al.^24^ found that there are moderate and weak correlations between vertical skeletal variables and soft tissue smile variables. According to the moderate correlations, as SN-GoGn and anterior facial height increases so does the interlabial gap while the smile index reduces. In addition, this study reported that along the y- and z-axis, the intercommissural width, smile index, and lower lip all have significant multiple regression models. Campbell et al.^16^ compared the range of movement in the smiling position between people with normal overjet and those with increased overjet, and they determined that the average movement of the landmarks in the normal overjet group is greater than in the increased overjet group.

#### Age

Two studies^20^^,^^21^ analyzed smile dimensions across age groups. Souccar et al.^20^ grouped the age of participant into five groups: 20 to 30, 30 to 40, 40 to 50, 50 to 60, and >60 years. The researchers reported that the upper lip length during smiling and the mouth width in the resting position increase significantly as age increases. By contrast, gingival display and the maxillary crown length during smiling decrease as age increases. According to the study by Tanikawa et al.,^21^ the mouth corners of the older group at rest and smile are narrower along the x-axis. The older group has lower-positioned cheeks and chin vertical positions during rest and smile, according to the y-axis. When the older group is at repose, their mouth corners and lower lips are situated in more inferior positions, and when they smile, their upper lips are situated in more inferior positions. Along the z-axis, when an older group is at rest and smiling, the nasal bridge and infraorbital areas are more protuberant along the z-axis. At rest, the older group has more retruded mouth corners. Moreover, they also concluded that the older group observes less soft tissue movement during the 3D facial changes in facial expression development and the transition from resting to smiling than the younger group.

#### Resting versus smiling position

Two studies analyzed the discrimination of resting versus smiling posture.^24^^,^^26^ Parra et al. compared the length of the alar base and the nasolabial angle between resting and smiling positions.^26^ The results revealed that the mean value of the nasolabial angle is unchanged, whereas the length of the alar base changes significantly in the smiling position. Tanikawa et al.^21^ analyzed the discrimination of resting versus smiling posture in the younger group and older group. They concluded that from rest to smile, the younger group has a large retrusive movement of the lip commissure and upper lip, a decrease in the labio-mental fold, a retrusive movement of the lower lip, a decrease in protrusion at the level of the lower lip and at the chin, and an increase in facial width. On the contrary, the older group has a significant decrease in lower lip height but increase in cheek protrusion, and retrusive movement of nasal ala.

#### Attractive score

One study by Li et al.^28^ compared the position of maxillary central incisors between the attractive smiling female sample and the ordinary smiling female sample. The measurements they analyzed were the anteroposterior distances from the maxillary central incisors (mFA) to four vertical lines. The result revealed the average maxillary incisor position of the attractive group was more posteriorly to all vertical lines than that position of the ordinary group. In addition, they concluded that the mFA to the vertical line passed through soft tissue subnasale (SSA) and the mFA to Glabella distances are esthetically important factors in female profiles.

## Discussion

This scoping review identified twelve studies published between 2010 and 2023 that involved the 3D analysis of a posed smile in adults. The included studies analyzed normal smiles in adults through 3D imaging and the following types of measurement: linear and angle measurement in the resting and smiling position, curving line, landmark distance ratio during smiling, landmark position, landmark displacement from resting to smiling, and the surface comparison method. According to our review, most of all studies contribute the same opinion that these techniques are completely non-invasive and non-contact imaging systems that offer superior accuracy, reliability, and speed compared to 2D photography.

Regarding the type of measurement, our results indicated that most of the included studies focused on linear and angle measurement in the resting and smiling positions, the landmark distance ratio during smiling, and landmark displacement from resting to smiling. Only two studies reported the landmark position, and three studies used the surface comparison method based on 3D meshes and the closet point-to-point distances. Because the lip framework is a critical smile component,^32^ the researchers that conducted linear and angular measurements between landmarks may not gain a comprehensive understanding of the morphology of soft tissues. Other techniques applied in soft tissue analysis have potential application in smile analysis. For example, Ayoub et al.,^33^ Sforza et al.,^34^ and Ferrario et al.^35^ conducted surface area and volume measurement at rest position only. In addition to the method that superimposed the 3D dental image with 3D facial soft tissue in the study of Masoud et al.,^23^ we could measure the lip thickness, which is an influent variable on the profile with respect to the position of the incisors.^36^ We, therefore, suggest conducting more research measuring the surface area, volume, and soft tissue thickness to examine smile morphology to fill the research gaps we discussed above.

The included studies involved subgroup comparisons in terms of sex, ethnicity, smile type, dental occlusion and skeletal pattern, and age, resting versus smiling position, as well as attractive score. We can determine that those subgroups are independent variables of their studies. Most of the included studies compared smiles between sexes, and a few studies compared smiles across ethnicities, smile type, ages, and attractive score. Rubin^6^ was the first to classify smiles into the three following types: Mona Lisa, canine, and full-denture smiles. Each type varies in terms of its direction and the strength of the individual perioral muscles. By contrast, Pucciarelli et al.^31^ evaluated the labial movement in terms of smile type. Thus, no study has analyzed and compared different smile types using 3D imaging techniques. For the dental occlusion and skeletal pattern subgroup, Campbell et al.^16^ was the only study that measured the magnitude of movement during smiling on the basis of different anteroposterior dimensions of occlusion between people with normal and increased overjet. No study has implemented another measurement for analysis by the type of overjet. In terms of age, Souccar et al.^20^ reported that the upper lip length during smiling and the mouth width in the resting position increase as age increases, whereas gingival and maxillary incisal display decrease with age. This result is in line with that of Sachdeva et al.^37^ in their 2D imaging study. Souccar et al.^20^ found no significant difference in the upper lip length at rest among age groups; by contrast, in their 2D imaging study, Dindaroğlu et al.^38^ noted an increased upper lip length at rest with increased age. From our review, we suggest comparing smiles in three dimensions across various overjet types, overbite types, horizontal skeletal patterns, ethnicities, smile types, and ages for the future search or even get the norm of smile characteristics according to those variables. If we could identify the average 3D smile measurements of normal dentoskeletal participants, we could then compare them with those who were graded with a high esthetic score of smiles using a questionnaire. This comparison would involve various measurements different from the study conducted by Li et al.,^28^ which primarily focused on the anteroposterior position of the maxillary central incisor. Since there is no study comparing smiles on the basis of different anteroposterior dimensions of occlusion, we might perform measurements for analysis between people with normal and irregular overjet or anteroposterior skeletal relationship. For the vertical dimension, we recommend analyzing the type of overbite as well. Furthermore, based on the classical studies of Hulsey^10^ and Ackerman et al.^8^ which compared the features of a posed smile between orthodontically treated patients and participants who were untreated with normal occlusion in 2D images, we might consider conducting another research study in a similar manner, but utilizing 3D imaging techniques to obtain more explicit results.

In addition, one study^27^ did not group the participants, but instead, they compared the mean facial proportions when smiling to the golden ratio (1.618). The study reported that out of the four observed ratios, three of them, which were horizontal ratios, were close to the golden ratio. However, this study only benefited from the 3D camera in terms of photo accuracy because their observed ratios were calculated from the 2D measurements projected onto three planes. Therefore, we could advance future research by comparing the other 3D measurements we previously suggested with the golden ratio.

In conclusion, we recommend conducting more research, including the following types of measurements: the surface area, volume, and soft tissue thickness, to examine smile morphology. Regarding the independent variables, we suggest studying the effects of various overjet types, overbite types, horizontal skeletal patterns, ethnicities, smile types, and ages on 3D smile characteristics. Moreover, 3D quantitative analysis of attractive smiles, orthodontically treated smiles, and other ratios associated with the golden ratio should be further investigated.

Our scoping review has some limitations. We could only include publicly available articles written in English. Therefore, relevant studies conducted and published in local journals or studies published in other languages were not included in the analysis.

## Declaration of competing interest

The authors have no conflicts of interest relevant to this article.
